# Impact of Innovative Treatment Using Biological Drugs for the Modulation of Diffuse Cutaneous Systemic Sclerosis: A Systematic Review

**DOI:** 10.3390/medicina59020247

**Published:** 2023-01-27

**Authors:** Diego Fernández-Lázaro, María Iglesias-Lázaro, Evelina Garrosa, Saray Rodríguez-García, David Jerves Donoso, Eduardo Gutiérrez-Abejón, Conrado Jorge-Finnigan

**Affiliations:** 1 Department of Cellular Biology, Genetics, Histology and Pharmacology, Faculty of Health Sciences, University of Valladolid, Campus of Soria, 42004 Soria, Spain; 2 Neurobiology Research Group, Faculty of Medicine, University of Valladolid, 47005 Valladolid, Spain; 3 Department of Medicine, Faculty of Health Sciences, University of Valladolid, Campus of Soria, 42003 Soria, Spain; 4 Internal Medicine Department of Soria University Assistance Complex (CAUSO), Santa Bárbara Hospital, Castile and Leon Health (SACyL), 42005 Soria, Spain; 5 Pneumology Department of Soria University Assistance Complex (CAUSO), Santa Bárbara Hospital, Castile and Leon Health (SACyL), 42003 Soria, Spain; 6 Department of Anatomy and Radiology, Faculty of Health Sciences, University of Valladolid, Campus of Soria, 42004 Soria, Spain; 7 Pharmacological Big Data Laboratory, Faculty of Medicine, University of Valladolid, 47005 Valladolid, Spain; 8 Pharmacy Directorate, Castile and Leon Health Council, 47007 Valladolid, Spain; 9 Dermatology Department of Soria University Assistance Complex (CAUSO), Santa Bárbara Hospital, Castile and Leon Health (SACyL), 42005 Soria, Spain

**Keywords:** scleroderma, systemic sclerosis, tocilizumab, belimumab, riociguat, abatacept, pulmonary function, skin disease, health status, safety

## Abstract

Scleroderma or systemic sclerosis (SSc) is an autoimmune disease affecting the connective tissue, characterized by fibrosis of the skin and internal organs. There is currently no curative treatment available, so therapeutic action is aimed at a symptomatic treatment of the affected organs. The development of biotechnology has made it possible to implement certain biological drugs that could represent a window of opportunity to modulate the evolution and symptomatology of scleroderma with greater efficacy and less toxicity than conventional treatments. This study aimed to review the current evidence critically and systematically on the effects of biological drugs on the pulmonary function, skin disease, and health status of patients afflicted by diffuse cutaneous systemic sclerosis (dcSSc). Three electronic databases (Pubmed, Dialnet, and Cochrane Library Plus) were systematically searched until the cut-off date of October 2022. The review was conducted following the Preferred Reporting Items for Systematic Reviews and Meta-Analyses (PRISMA) guidelines and included original articles in English and Spanish with a controlled trial design, comparing biological drug treatments (tocilizumab, belimumab, riociguat, abatacept, and romilkimab) with a control group. The methodological quality of the studies was assessed using the McMaster quantitative form and the PEDro scale. A total of 383 studies were identified, 6 of them met the established criteria and were included in the present systematic review. A total of 426 patients treated with tocilizumab, belimumab, riociguat, abatacept, and romilkimab were included. The results showed substantial non-significant (*p* < 0.05) improvement trends after treatment with the biological drugs included in this review for the modified Rodnan Scale Value, Forced Vital Capacity, and Carbon Monoxide Diffusion Test; however, no benefits were shown on the Health Assessment Questionnaire–Disability Index when compared to the control group. Biological drugs, therefore, maybe a new therapeutic strategy for dcSSc and could be recommended as an additional and/or adjunctive treatment that promotes anti-fibrotic activity. This review could further define the clinical rationale for the use of biologics in the treatment of dcSSc and could provide key details on the study protocol, design, and outcome reporting.

## 1. Introduction

Scleroderma refers to a heterogeneous group of autoimmune fibrosing disorders. Etymologically, it is derived from Greek and means “hard skin” (*skleros*: hard; *dermis*: skin) [1]. Scleroderma was first described in 1752 by the Italian physician Carlo Curzio as a disease that “*transformed the skin into wood*” [2]. Generalized scleroderma or systemic sclerosis (SSc) is an autoimmune disease that affects the connective tissue, characterized by skin and organ fibrosis (heart, lungs, and kidneys), generalized microvasculopathy, and antibody responses against various cellular antigens and alterations in the immune system [3]. Clinically, two subtypes can be distinguished: (i) limited cutaneous systemic sclerosis (lcSSc), which progresses slowly by hardening skin in the acral areas, distal to elbows and/or knees, and on the face; (ii) diffuse cutaneous systemic sclerosis (dcSSc) which progresses rapidly by a thickening of the trunk and/or limb proximal regions (Table 1), [1]. Severe and serious affections occur in some organs (mainly in the lungs, heart, and kidneys), and has a poor prognosis [3]. In this way, Sulli et al. [4] demonstrated that blood perfusion is significantly lower in patients with SSc than in healthy subjects. The most affected parts were the fingertips, periungual, and palm areas, but not on the face or back of the hands, highlighting a selective affectation of the microcirculation due to the damage caused by SSc. These investigators used laser speckle contrast analysis (LASCA), which was an innovative safe technique to quantify blood perfusion (BP) in different areas of the body [4].

Currently, the annual incidence is 1 to 20 cases per million per year [10], with variations found depending on the geographical area, which may suggest that the rate of occurrence of new cases in the susceptible, at-risk population is conditioned by a genetic predisposition, individual hormonal behavioral determinants, and exposure to environmental factors [10,11,12]. The incidence is higher in the United States than in Europe, with a higher prevalence in African Americans than Caucasians [5]. SSc is more common in women than men, at a ratio of 3–5 women to every man, with the highest incidence rates between the ages of 30 and 50 years [5]. No gender differences were observed in vascular or gastrointestinal involvement; however, tendon rubbing or forced vital capacity (FVC) <70% occurs more commonly in men than in women. Regarding the age of mortality, it does not differ significantly between the sexes. Notwithstanding, for the male group, the development of symptoms and mortality is higher [12]. A positive correlation has been observed between different major histocompatibility complex (MHC) class II haplotypes and certain antibody subtypes (anti-centromere antibodies (ACA), HLA-DQB1*0501 and anti-topoisomerase) rarely found in healthy people or other connective tissue diseases [13,14]. Similarly, several alleles (HLA-DRB1*1501, DRB1*0701, DQA1*0102, DQB1*0602) have been described that could have a protective effect against SSc, as they have been found to be decreased in patients with the disease. The same polymorphism in the PTPN22 gene, associated with other autoimmune diseases, is also linked to SSc [15]. Exposure to silica, vinyl chloride, resins, and organic solvents and infections, cytomegalovirus, and parvovirus B19 are all known to trigger SSc in susceptible individuals [5].

The preliminary classificatory criteria for the classification of SSc were developed in 1980 by the American College of Rheumatology (ACR); advances in diagnostic tools and a joint effort by the ACR and the European League Against Rheumatism (EULAR) in 2013 allowed them to be expanded, giving them greater sensitivity and specificity, by including patients with early disease or very limited skin involvement (Table 2) [16]. In relation to laboratory findings, more than 95% of patients with SSc develop positive antinuclear antibodies (ANA), with ACA and anti-polymerase I being the most frequent, although up to seven specific ANA have been described; with anti-Scl70, anti-Th/To, and anti-U3RNP being those associated with a worse patient prognosis [17]. Other complementary tests include: radiodiagnostics for the detection of calcinosis; respiratory function tests assessing (FVC and carbon monoxide diffusion test (DLCO)); Doppler echocardiograms, electrocardiograms, and a Holter monitor for the study of cardiac involvement; barium esophageal study, esophageal manometries, breath tests and digestive endoscopies for digestive tract involvement; in addition, arterial monitoring and a Doppler ultrasound are used to assess renal involvement [18,19].

There is currently no curative or disease-modifying treatment available. Moreover, the loss of efficacy over time over long periods of treatment and with significant side effects has been demonstrated. Thus, therapeutic action will be aimed at symptomatic treatments of the affected organs according to their severity, depending on the evolution and duration of the disease [20]. Therefore, the need for an individualized precision pharmacological regimen seems obvious. To this end, therapeutic algorithms have been developed that provide for general measures such as first-, second-, and third-line drug regimens (Table 3) [20,21]. The Janus kinase (JAK) signaling pathway is an area of emerging interest in dermatology. In fact, recently, Moriana et al. [22] reported that JAK inhibitors could represent a safe and effective treatment option for cutaneous fibrosis and interstitial lung disease (ILD) in SSc. Additionally, the combination of two immunosuppressants, rituximab plus methotrexate, had potential efficacy for the skin and stabilization of internal organ involvement, and on some microangiopathies in early dcSSc [3]. In recent decades, the development of biotechnology has made it possible to implement hundreds of biological drugs, which are large protein molecules produced by living organisms that modulate the course of a disease by acting on a specific target. Biological drugs include hormones, monoclonal antibodies, blood products, immunomodulators, and vaccines [23]. According to the European Medicines Agency (EMA), a biosimilar is a biological drug that contains a version of the active substance of an original biological product or reference product whose patent has expired [24]. Biological drugs may represent a window of opportunity to modulate the progression and symptomatology of SSc with greater efficacy and lower toxicity than conventional treatments [20] (Table 4). Therefore, the purpose of this study was to review the current evidence critically and systematically on the effects of biological drugs on the health biomarkers of patients afflicted by dcSSc. The review protocol is published in the Prospective Registry of Systematic Reviews (PROSPERO); reference CRD42023387373.

## 2. Materials and Methods

### 2.1. Search Strategy

This systematic review was conducted following the specific methodological guidelines of the Preferred Reporting Items for Systematic Review and Meta-Analyses (PRISMA) [26] and the PICO question model for the definition of the inclusion criteria: P (population): “dcSSc’s patients”; I (intervention): “treatment with biological drugs”; C (comparison): “same conditions with placebo, sham therapy or no intervention or pre/post comparison data group”; O (outcomes): “Skin disease (modified Rodnan scale value [mRSS]); pulmonary function test (forced vital capacity [FVC] and carbon monoxide diffusing capacity [DLCO]); and health status (Health Assessment Questionnaire [HAQ] Disability Index [DI] → HAQ-DI Scale). These parameters were included as outcomes as they are commonly investigated in health biomarker studies and in SSc research [27].

A structured search was carried out in the electronic databases: Medline (PubMed), Dialnet, and Cochrane Library Plus between September 2022 and December 2022. Publications from the last 5 years were included, given the evolution of the research in biological treatments for autoimmune diseases. Search terms included a mix of medical subject headings (MeSH) and free text words for key concepts related to biological drugs and SSc: scleroderma, systemic sclerosis, scleroderma diffuse, diffuse cutaneous systemic sclerosis, therapeutics, tocilizumab, biological therapy, antibodies, and monoclonal (monoclonal antibodies); all linked using the Boolean operators OR and AND. The complete search strategy is included in Appendix A. The review was carried out completely independently —titles, abstracts, and full texts—by two investigators (D.F.-L. and M.I.-L.). In addition, the inclusion criteria were independently evaluated and the disagreements generated were resolved by another reviewer (C.J-F.). There were no additional records of reference lists of the relevant articles or gray literature.

### 2.2. Inclusion and Exclusion Criteria

To select the studies, the following inclusion criteria were applied: (i) adults with the condition dcSSc; (ii) studies evaluating the effect of biological drugs accepted for the treatment of dcSSc in humans (excluding animal and/or in vitro studies); (iii) clinical trials, randomized and non-randomized trials, and pre-test/post-test designed studies (excluding reviews, notes, and other-than-original studies); (iv) studies evaluating outcomes (primary or secondary) of skin, respiratory, and functional capacity biomarkers; (v) studies that clearly report the dose, frequency, and route of drug administration; (vi) languages were restricted to English and Spanish; (vii) articles of methodological quality ≥ 11 points according to the McMaster University Occupational Therapy Evidence-Based Practice Research Group for quantitative studies [28] and ≥9 points according to the Physiotherapy Evidence Database (PEDro) scale [29]. Records that did not meet the criteria were excluded from this systematic review.

### 2.3. Methodological Quality Assessment

The methodological quality of the articles was assessed using the McMaster University Occupational Therapy Evidence-Based Practice Research Group [28] and the PEDro scale [29] as tools designed to assess the methodological quality of clinical designs.

### 2.4. Data Extraction

The data of the selected studies were summarized in Table 5. The following information was included: name of the first author, year of publication, country where the study was conducted, study design, sample size, gender and age of the participants, duration of the intervention, dose, and mode of administration of the treatment. This was performed by two study investigators (D.F.-L. and M.I.-L.) and disagreements were resolved by the intervention of another study investigator (C.J.-F.).

## 3. Results

### 3.1. Selection of Studies

**Figure 1:**
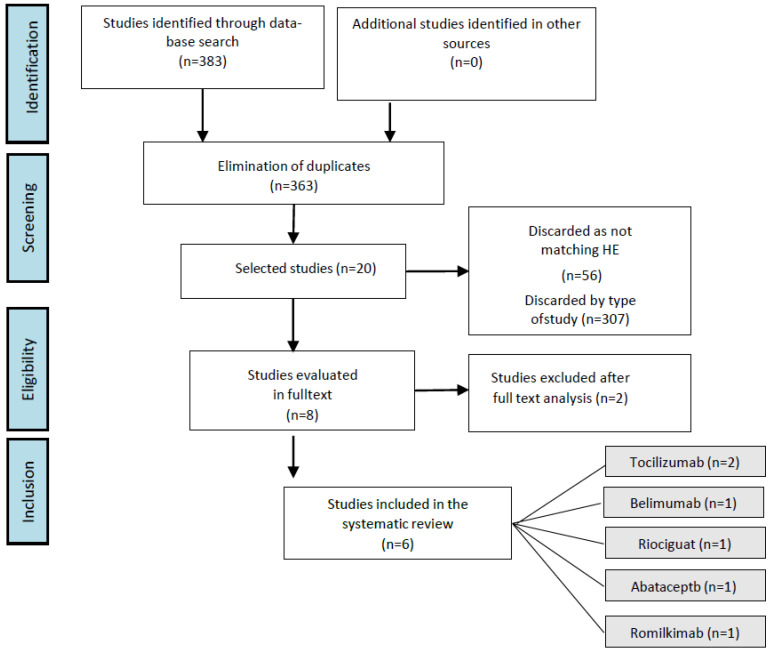
Flow chart depicting the processes of identifying and selecting relevant studies according to the Preferred Reporting Items for Systematic Reviews and Meta-Analyses (PRISMA) guidelines.

We initially identified a total of 383 records. Among them, 20 duplicates were eliminated, 307 were not selected by study type, and 56 were not related to the objective of the study (systemic scleroderma). Two articles were also excluded after a full text review. The reasons for the exclusions after the full text review were inadequate results, because these studies were not related to the results evaluated in this study as skin disease, pulmonary function, and health status, and the remaining six studies [30,31,32,33,34,35] met our inclusion criteria and were included in the present systematic review (Figure 1).

### 3.2. Methodological Quality Assessment

One study was considered of “good quality” [31], one of “very good quality” [32], and four studies were considered of “excellent quality” [30,33,34,35], according to McMaster [28] (Table A1) For the PEDro scale [29], the score was 10 points for two studies [32,33] and 11 points for four studies [30,31,34,35], corresponding to “very good” and “excellent” quality, respectively (Table A2). No study was excluded for not reaching the minimum quality threshold (Table A1 and Table A2).

### 3.3. Characteristics of the Participants and Interventions

The total number of dcSSc patients included at the baseline in the studies was 426. All participants were ≥18 years old diagnosed with dcSSc according to the 1980 ACR or 2013 ACR/EULAR diagnostic criteria [16], with an active disease. In the review, we included six studies [30,31,32,33,34,35] that analyzed biological drugs: tocilizumab [32,35], with 2 different doses as 162 mg/week [32] and 8 mg/kg/month [35]; belimumab (10 mg/kg + 1000 mg twice/day mycophenolate mofetil [31]; riociguat (0.5–2.5 mg 3 times a day) [34]; abatacept (125 mg once a week) [33]; and romilkimab (200 mg/week) [30]. No study included patients of more than 5 years of evolution since the first symptom, excluding Raynaud’s phenomenon (RP) (Table 5).

One study excluded patients who had previously received mycophenolate mofetil > 3 months, rituximab or belimumab; or if they required >10 mg/day of prednisone [31]. Khanna et al. [34] excluded patients receiving nitrates, phosphodiesterase inhibitors, or SSc-specific treatments. Additionally, Shima et al. [35] excluded all patients who had used any biological drug in the previous 6 months. In the abatacepts study conducted by Khanna et al. [33], patients receiving immunomodulatory therapy were excluded. However, Allonote et al. [30] included patients on stable low-dose immunosuppressive therapy plus romilkimab.

### 3.4. Outcome Evaluation

A schematic summary was made of the different studies selected, organizing it into author(s) and year, study design, population, intervention, parameters analyzed, and main conclusions (Table 5).

### 3.5. Measure for Skin Disease

All studies (30–35) included in this systematic review evaluated the effectiveness of biological drugs on skin fibrosis using the mRSS scale. Only romilkimab [30] showed a significant decrease (*p* < 0.05) in induration compared to the control group. Tocilizumab [32,35], belimumab [31], riociguat [34], and abatacept [33] showed notable trends in the improvement on skin hardening.

### 3.6. Pulmonary Function Test

Lung functioning was assessed by FVC (30–34) and DLCO [30,31,32,34,35], with non-significant (*p* > 0.05) improvements in FVC being reported after the administration of belimumab [31], riociguat [34], romilkimab [30], and abatacept [33] when compared with the control group, and these increases in FVC were significant (*p* < 0.05) in belimumab-treated patients from the baseline to the end of the intervention [31]. DLCO showed a slight increase, yet non-significant (*p* > 0.05), in those patients treated with tocilizumab [32], belimumab [31], riociguat [34], and romilkimab [30] when compared to a control group. Belimumab treatment [31] showed significant (*p* < 0.05) increases in DLCO when compared with a baseline. On the contrary, Khanna et al. [32] reported non-significant (*p* > 0.05) decreases in the tocilizumab intervention group relative to the control and to the end of intervention after 48 weeks of treatment for both lung biomarkers FVC and DLCO.

### 3.7. Health Status

The alternative disability index assessed by the HAQ-DI scale was described in four studies included in this systematic review [30,32,33,34]. Treatments with riociguat [34], romilkimab [30], and abatacept [33] showed moderate non-significant decreases (*p* > 0.05) in the HAQ-DI scale with respect to the control group, while treatment with tocilizumab [32] did not induce any change when compared to the control group. Non-significant decreases (*p* > 0.05) also have been reported in the tocilizumab [32], romilkimab [30], and abatacept [33] intervention groups when comparing to the baseline. These results were contrary to those reported after 52 weeks of treatment with riociguat [34].

### 3.8. Safety

Adverse events were less reported in the intervention group compared to the control group in three studies (30,31,33), and more were reported in the tocilizumab and riociguat studies [32,34]. However, adverse events of severity were much lower in all five studies in the intervention group than in the control group. The most reported adverse effects in those patients were infections, followed by respiratory and gastrointestinal disorders (Table 6).

## 4. Discussion

The purpose of this systematic review was to critically study the impact of biological drug effects on the health biomarkers of patients afflicted by dcSSc. Six registers met the pre-specified inclusion/exclusion criteria. Overall, improvements have been reported regarding skin disease (mRSS) and pulmonary function parameters (FVC and DLCO), after treatment with the biological drugs included in this review. Yet, no benefits were shown on health status (HAQ-DI), and on the other hand, adverse effects associated with the use of biological drugs such as infections, respiratory, and gastrointestinal disorders may occur. Due to the differently measured results in the studies, the following results were divided into different sections to provide a clearer analysis.

### 4.1. Skin Disease

For a correct diagnosis, the thickening of the skin must be evaluated through the histological characteristics of the skin biopsy sample, adding subcutaneous tissue and muscle fascia. This type of skin biopsy allows analysis of the eosinophilic infiltrate, lymphocytes, and plasma cells [36]. However, the pathogenesis of dcSSc is complex and not completely understood, although there is evidence of the role of T and B lymphocytes in the production of profibrotic cytokines and in the activation of fibroblasts [37]. One of the cytokines implicated in the pathogenesis of dcSSc is interleukin 6 (IL-6), which is found in high concentrations in the skin and serum of patients with dcSSc [38], which in turn correlates with the severity of the disease due to the fact that IL-6 is related with high mRSS scores that represent the degree of skin involvement [38]. The results for treatment with tocilizumab [32,35] shows an improvement in dermal hardening. These results were consistent with those described by Denton et al. [39], which reported how protein production, migration, and contractility were reduced after 24 weeks of treatment with tocilizumab (162 mg) to an in vitro assay with dermal fibroblasts isolated from dcSSc patients, due to a blockade of interleukin-6 (IL-6) α-receptors.

The selective immunosuppressive action of Belimumab [31] on B lymphocytes significantly decreased skin hardening in dcSSc patients. This biological drug was also used to reduce skin hardening in patients with cutaneous lupus erythematosus [40]. The treatment with riociguat [34] and abatacept [33] also tended to decrease skin induration, while romilkimab [30] decreased dermal fibrosis significantly by binding and neutralizing IL-4 and IL-13, directly by fibroblast activation, and indirectly by stimulating transforming growth factor beta (TGF-β); adding on top of that, the direct effect of IL-4 on T-cell activation triggers fibroblast activation and drives collagen synthesis, as well as anti-topoisomerase-1 antibodies [41].

These results in skin alterations would encourage the use of biological drugs not only due to their capability of counteracting and balancing skin hardening, but stimulating non-fibrotic tissues in patients with dcSSc.

### 4.2. Pulmonary Involvement

Pulmonary disease is present in most patients with SSc and is currently the leading cause of mortality. Two types of damage predominate in these patients: interstitial lung disease and pulmonary arterial hypertension [42]. It is acknowledged that patients with SSc often demonstrate a restrictive pattern, with reduced forced FVC and DLCO. Therefore, the improvement of respiratory parameters is an indirect measure of the efficacy and of the pharmacological effect in SSc because it would reduce the thickening, rigidity, and scarring of the respiratory membrane, composed of the alveolar endothelium and the vascular endothelium, and the basement membrane that they both share [43]. In three studies [30,31,33] included in this systematic review with romilkimab [30], abatacept [33], and belimumab [31], there was a notable tendency to improve FVC and DLCO, while in one study of riociguat [34], the amelioration was only evidenced by FVC. The results of a network meta-analysis show that no treatment (cyclophosphamide, mycophenolate, cyclophosphamide plus high-dose prednisone, cyclophosphamide followed by azathioprine, rituximab, pirfenidone, nintedanib, and pomalidomide) influenced DLCO and only rituximab significantly decreased FVC compared to a placebo [44]. In addition, another study reported that cyclophosphamide plus azathioprine and mycophenolate did not significantly reduce the decline in FVC [45]. Improvements in DLCO would be especially important as it would indicate the restoration of the thickness of this lung membrane, which allows oxygen and carbon dioxide to pass through it, avoiding hypoxemia, which is one of the characteristic features of interstitial lung diseases, such as pulmonary fibrosis developed by dcSSc.

In addition, the benefits on DLCO could mean a restoration of blood flow problems, caused by an increase in the thickness of the respiratory membrane or a concomitant pulmonary vascular disease, which would improve the rate of gas transfer, by unblocking the circulation and increasing the opportunities for gas displacement. However, after the administration of tocilizumab [32,35], no benefits have been observed on pulmonary fusion monitoring parameters such as FVC or DLCO, results that are consistent with those obtained by Manfredi et al. [46] in rheumatoid arthritis with interstitial lung disease. Perhaps the decrease in IL-6 would be insufficient to stop fibrosis of the respiratory membrane. Therefore, romilkimab, abatacept, and belimumab would be the treatments with the potential to attenuate, modulate, and control respiratory disturbances in patients with dcSSc. This would position these drugs, due to the benefit they induce as biological therapies, as targeted anti-fibrotic treatments that would complete the traditional pharmacological management of combinations of immunosuppressants, particularly cyclophosphamide and mycophenolate mofetil.

### 4.3. Health Status

Potentially, the improvement in quality of life is based on the control of tissue fibrosis: (i) respiratory, because it would increase respiratory capacity; and (ii) dermal, which allows for a greater range of movement [47]. Symptomatic improvements would stimulate the performance of activities of daily living [48]. Although, in general, we have described in this review the mitigation of pulmonary and respiratory symptoms for patients treated with biological drugs, no HAQ-DI improvements have been obtained [30,31,32,33,34,35]. This could be because the improvements described are insufficient to improve the eight aspects of daily life during the last week (dressing and grooming, getting up, eating, walking, hygiene, reaching, grasping, and activities) assessed by the HAQ-DI [49]. Two important limitations must also be taken into account: the HAQ-DI is a self-perceptive health assessment questionnaire developed for patients with rheumatoid arthritis, whose adaptation for dcSSc has little correlation with the severity of the disease [46].

### 4.4. Safety

The use of biological therapies raises concerns due to the lack of knowledge about the long-term effects of blocking or the persistent inhibition of cytokines, or their receptors, or of immune cells that play a key role in their mechanisms [48]. Belimumab [31] was the biological treatment that induced the most adverse effects. These results are consistent with those reported after the administration of belimumab in patients with lupus erythematosus [50] with infections, especially of the upper respiratory tract, being the most common adverse effects. Comparatively, these side effects are less than the main adverse events of JAK inhibitors. Some of the side effects of JAK inhibitors were: infections, gastrointestinal disorders, elevated liver enzymes, and dyslipidemia, but they do not lead to the discontinuation of treatment [22].

### 4.5. Clinical Trials of Biological Drugs for the Modulation of Diffuse Cutaneous Systemic Sclerosis

Some clinical trials have been developed to show more evidence to incorporate biological drugs into a routine and/or care protocols. Roche Pharma (Basel, Switzerland) announced in 2021 that the US Food and Drug Administration (FDA) has approved Actemra/RoActemra^®^ (tocilizumab) for subcutaneous administration to slow the rate of decline in lung function in adult patients with interstitial lung disease associated with SSc. Actemra/RoActemra was the first FDA-approved biologic therapy for the treatment of this disease. The FDA approval was based on data from the focusSced (ClinicalTrials.gov Identifier: NCT02453256.phase III) randomized, double-blind, placebo-controlled clinical trial conducted in 212 adults with SSc. Information from the fascinating (ClinicalTrials.gov Identifier: NCT01532869), phase II/III, randomized, double-blind, placebo-controlled clinical trial in patients with SSc was also used [44].

A recent clinical trial (ClinicalTrials.gov Identifier: NCT03844061), which will run until June 2024, at the Hospital for Special Surgery (New York, NY, USA) with the pharmaceutical GlaxoSmithKline (Bretford, UK) as a collaborator, is using a combination therapy of belimumab and rituximab (monoclonal antibody) for the treatment of SSc. It is hypothesized that a combination therapy of rituximab and belimumab with mycophenolate mofetil background therapy will improve SSc skin fibrosis compared to a placebo and mycophenolate mofetil treatment in a group of early SSc patients [51]. A randomized, double-blind, placebo-controlled phase II study to investigate the efficacy and safety of riociguat (Adempas, BAY63-2521) in patients with dcSSc (ClinicalTrials.gov Identifier: NCT02283762), sponsored by Bayer (Berlin, Germany) showed remarkable results in mRSS and the HAQ-DI [52].

Bristol-Myers Squibb (New York, NY, USA) launched a pilot study to evaluate the safety and efficacy of abatacept in patients with systemic sclerosis (ClinicalTrials.gov Identifier: NCT00442611) in collaboration with Stanford University (Stanford, CA, USA). The investigators have observed a marked clinical improvement after abatacept therapy in patients with SSc that was associated with the modulation of inflammatory pathways in the skin [53]. Despite the good preliminary results of romilkimab in relieving the symptoms of CSS and slowing its progression, on 9 January 2020, the European Commission granted orphan designation EU/3/19/2246 to Sanofi-Aventis Groupe, (Paris, France), for romilkimab for the treatment of SSc. Romilkimab was withdrawn from the Union Register for orphan drugs in March 2021 at the request of the sponsor [54].

### 4.6. Strengths and Limitations

The authors of this review acknowledge some limitations. First, a limited number of manuscripts met the inclusion criteria. Nevertheless, our systematic approach followed the PRISMA method [26] and the search was conducted using three electronic databases (Pubmed, Dialnet, and Cochrane Library Plus). The McMaster methodological quality assessment tool [28] and the PEDro scale [29] were used to ensure that all selected records met the minimum quality criteria, and a number of outcomes commonly used in chronic disease drug research were included. Further, our systematic review was registered in the PROSPERO (CRD42023387373) public database. Secondly, there is a large heterogeneity of studies in terms of outcomes, dosage of supplements, and duration of intervention that justifies caution in interpreting the results.

## 5. Conclusions

Biological drugs have the potential to provide a therapeutic alternative as an add-on and/or adjunctive treatment to biological drugs with anti-fibrotic activity. Considering the results included in this systematic review (MRSS, FVC, DLCO), biologics would potentially improve tissue function, highly correlated with survival, although no benefits over HAQ-DI have been reported, or severity of adverse effects. This review could further define the clinical rationale for the use of biologics in the treatment of dcSSc and could provide key details on the study protocol, design, and outcome reporting. However, further studies on the treatment of dcSSc with biologics are required.

## Figures and Tables

**Table 1:** Clinical signs, prevalence, and symptomatology in diffuse cutaneous systemic sclerosis (dcSSc).

Involved Organ(s)	Clinical Signs		Prevalence	Symptomatology
Vascular [4,5]	Raynaud’s phenomenon		100%	Changes in skin coloration
	Digital ulcers		41.6%	Hard-to-heal lesions → infection, osteomyelitis, gangrene
Cutaneous [6]	Dermal hardening		100%	
	Pigmentation alterations			Patchy areas of depigmentation.
	Calcinosis cutis			Subcutaneous nodules Pain
Musculoskeletal [5]	Fibrosis		46–97%	Contractures Morning stiffness
	Arthritis			Arthralgias
Gastrointestinal [7]	Esophageal hypomotility		90%	Dysphagia
	Gastroesophageal reflux			Bacterial overgrowth
	Stomach hypomotility			Delayed emptying → Premature satiety, fullness, bloating, nausea
	Intestinal hypomotility			Chronic intestinal pseudo-obstruction Malabsorption
	Anorectal dysfunction			Incontinence
Renal [8]	Renal scleroderma crisis		10%	Malignant high blood pressure Angiopathic hemolytic anemia Thrombocytopenia Proteinuria Macrohematuria Acute renal failure
Cardiac [9]	Arrhythmias	Ventricular	90%	Fatigue Palpitations Syncope Dizziness
		Supraventricular	66%	
		Ventricular multiform premature beats	40%	
		Left bundle branch block	16%	
		First-degree atrioventricular blocks	8%	
	Pericardial conditions	Pericardial effusion	78%	Chest pain Dyspnea Fever
		Pericarditis	77.5%	
	Myocardial dysfunction	RV dysfunction	69%	Fatigue Calf edema Dyspnea Venous congestion
		LV dysfunction	46%	
		Cardiac failure	20–25%	
	Valvular dysfunction	Mitral valves prolapse	20%	Dyspnea Pain Edema Palpitations Fatigue
Pulmonary [2]	Pulmonary interstitial disease		40%	Dyspnea Dry cough Crackles Pulmonary arterial hypertension *Cor pulmonale*
	Pulmonary arterial hypertension		15–20%	RV hypertrophy ↓ Cardiac output Heart failure

Abbreviations: RV: right ventricle; LV: left ventricle; ↓: decrease; →: produces.

**Table 2:** ACR/EULAR 2013 Systemic Scleroderma classification criteria. Adapted from Van Den Hoogen et al. [16].

Items	Sub-Item(s)	Weight/Score
Skin thickening of the fingers of both hands extending proximal to the metacarpophalangeal joints (sufficient criterion)		9
Skin thickening of the fingers (only count the higher score)	Puffy fingers	2
	Sclerodactyly of the fingers (distal to the metacarpophalangeal joints but proximal to the interphalangeal joints)	4
Fingertip lesions (only count the higher score)	Digital tip ulcers	2
	Fingertip pitting scars	3
Telangiectasia		2
Abnormal nailfold capillaries		2
Pulmonary arterial hypertension and/or interstitial lung disease (maximum score is 2)	Pulmonary arterial hypertension	2
	Interstitial lung disease	2
Raynaud’s phenomenon		2
SSc-related autoantibodies (anticentromere, anti-topoisomerase I [anti–Scl-70], anti-RNA polymerase III) (maximum score is 3)	Anticentromere	3
	Anti–topoisomerase I	3
	Anti–RNA polymerase III	3

Abbreviations: SSc: systemic sclerosis. These criteria are applicable to any patient considered for inclusion in an SSc study. The criteria are not applicable to patients with skin thickenings paring the fingers or to patients who have a scleroderma-like disorder that better explains their manifestations (e.g., nephrogenic sclerosing fibrosis, generalized morphea, eosinophilic fasciitis, scleredema diabeticorum, scleromyxedema, erythromyalgia, porphyria, lichen sclerosis, graft-versus-host disease, diabetic cheerio arthropathy). The total score is determined by adding the maximum weight (score) in each category. Patients with a total score of 9 are classified as having definite SSc.

**Table 3:** Therapeutic strategies used in the treatment of systemic sclerosis (SSc).

Affectation		General Measures	1st Line Treatment	2nd Line Treatment	3rd Line Treatment	New Therapeutic Tools
Peripheral vascular [4,5]		Avoid exposure to cold, sudden changes in temperature, stress, smoking, vasospastic substances.	Calcium channel blockers	Phosphodiesterase-5 inhibitors	If severe RP: Prostanoids	Prostacyclins and prostaglandins (Iloprost, Treprostanil) Statins Topical agents Biologic drugs (Riociguat, Bosentan) Botulinum toxin
					If mild RP: Calcium channel blockers or angiotensin receptor blockers. Skin involvement	
Skin [5]	mRSS ≤ 32	Avoid friction and trauma	Methotrexate	Mycophenolate mofetil		Pirfenidone Nintendanib Rituximab
	mRSS > 32		Mycophenolate mofetil	Methotrexate	Cyclophosphamides	Hematopoietic cell transplantation Pirfenidone Nintendanib Rituximab
	DU prevention		Calcium channel blockers	Phosphodiesterase-5 inhibitors		Bosentan Prostaglandins
	DU treatment		Calcium channel blockers	Phosphodiesterase-5 inhibitors	Prostanoids	
Musculoskeletal [5]		Moderate exercise and physiotherapy	Methotrexate	Low dose Corticosteroid	Hydroxychloroquines	Rituximab Tocilizumab Abatacept
Gastrointestinal [7]	↓ Motility	Prevent malnutrition	Proton pump inhibitors	Agents that high motility	Surgical treatment	
	Bacterial overgrowth		Antibiotherapy			
Renal [25]			Angiotensin-converting enzyme inhibitors	Calcium channel blockers Aldosterone II receptor antagonists	Renal replacement therapy Dialysis	
Renal Crisis [25]					Renal replacement therapy Dialysis	
Cardiac [9]	Arrhythmias		Verapamil Amiodarone	Ablation Pacemaker		
	Pericardial involvement		Anti-inflammatory drugs Corticosteroids	Pericardial drainage Pericardiocentesis		
	Myocardial dysfunction		Angiotensin-converting enzyme inhibitors Beta-blockers Diuretics	Cardiac resynchronization		
	Valvular dysfunction		Cardiac transplantation Transplantation of affected valves			
Pulmonary [2]		Mycophenolate mofetil	Lung transplantation			Rituximab Tocilizumab

Abbreviations: RP: Raynaud’s phenomenon; MmRSS: modified Rodnan scale; DU: digital ulcers.

**Table 4:** Function and mechanism of the biological drugs used for the treatment of systemic sclerosis (SSc) included in this systematic review.

Drug	Function	Mechanism
Tocilizumab	Immunosuppressant	It binds to soluble and membrane-bound IL-6 receptors and inhibits IL-6-mediated signaling
Belimumab	Selective immunosuppressant	IgG1 monoclonal antibody that specifically binds to the soluble form of human B-cell-stimulating protein
Riociguat	Anti-hypertension pulmonary	Stimulator of the soluble guanylyl cyclase
Abatacept	Selective immunosuppressant	Selectively modulates a key co-stimulatory signal for the full activation of T lymphocytes expressing CD28
Romilkimab	Selective immunosuppressant	Biospecific antibody to IgG4 which neutralizes IL-4 and IL-13

Abbreviations: IgG: immunoglobulin G; IL: interleukin.

**Table 5:** Studies included in the systematic review of the effect of biological drugs on health biomarkers in patients afflicted by diffuse cutaneous systemic sclerosis.

First Author, Year of Publication, Country, and Drug	Study Design	Participants (Baseline Sample Size, Age, Sex, Withdrawals, and Final Group Sample Size)	Intervention	Outcomes	Results
Allanore et al., 2020 France [30] *Romilkimab*	Phase II, randomized, double-blind, placebo-controlled clinical study	20 ♂ and 77 ♀ >18 years Study withdrawals: 10 *n_o_* = 48 Romilkimab *n_o_* = 49 PBO	200 mg Romilkimab or PBO SC/week for 24 weeks.	mRSS FVC% DLCO% HAQ-DI	GI vs. GC: ↓* mRSS ↑ FVC % ↑ DLCO % ↓HAQ-DI score Changes from baseline: ↓* mRSS ↓ FVC % ↓ DLCO % ↓HAQ-DI score
Gordon et al., 2018 USA [31] *Belimumab*	Randomized, double-blind, placebo-controlled pilot study	*n* = 20 >18 years Study withdrawals: 2 *n_o_* = 7 Belimumab + MMF *n_o_* = 10 PBO + MMF *n* = 6 Belimumab + MMF *n* = 9 PBO + MMF	10 mg/kg Belimumab or PBO c/2 weeks first 3 doses and c/4 weeks until week 48 + 1000 mg 2 times/day MMF 48 weeks	mRSS FCV% DLCO%	GI vs. GC: ↓ mRSS ↑ FVC % ↑ DLCO % Changes from baseline: ↓* mRSS ↑* FVC % ↑* DLCO %
Khanna et al., 2018 USA [32] *Tocilizumab*	Phase II, randomized, double-blind, placebo-controlled clinical trial	20 ♂ and 67 ♀ >18 years Study withdrawals: 36 *n_o_* = 44 PBO *n_o_* = 43 TCZ *n_o_* = 31 PBO-TCZ *n_o_* = 30 TCZ-TCZ *n* = 24 PBO-TCZ *n* = 27 TCZ-TCZ	162 mg TCZ sc 48 weeks double blind + 162 mg TCZ sc 48 weeks open period	total Mrss HAQ-DI score FCV% DLCO %	GI vs. GC: ↓ total mRSS ↔ HAQ-DI score ↓ FVC% ↓ DLCO % Changes from baseline: ↓ total mRSS ↓ HAQ-DI score ↓ FVC % ↓ DLCO %
Khanna et al., 2020 USA [33] *Abatacept*	Phase II, randomized, double-blind, placebo-controlled clinical study	22 ♂ and 66 ♀ >18 years Study withdrawals: 12 *n_o_* = 44 Abatacept *n_o_* = 44 PBO *n* = 35 Abatacept *n* = 34 PBO	125 mg Abatacept or PBO SC 1 times/week 12 months	mRSS FVC% HAQ-DI	GI vs. GC: ↓ mRSS ↑ FVC % ↓HAQ-DI score Changes from baseline: ↓ mRSS ↓ FVC % ↓ HAQ-DI score
Khanna et al., 2020 USA [34] *Riociguat*	Phase II, randomized, double-blind, placebo-controlled, international, multicenter clinical trial	29 ♂ and 92 ♀ >18 years Study withdrawals: 33 *n_o_* = 60 Riociguat *n_o_* = 61 PBO *n* = 42 Riociguat *n* = 46 PBO	0.5–2.5 mg Riociguat 3 times/day 52 weeks	mRSS FVC% DLCO% HAQ-DI	GI vs. GC: ↓ mRSS ↑ FVC % ↑ DLCO % ↓HAQ-DI score Changes from baseline: ↓ mRSS ↓ FVC % ↓ DLCO % ↑ HAQ-DI score
Shima et al., 2018 Japan [35] *Tocilizumab*	Randomized, open-label clinical trial	10 ♂ and 3 ♀ 20–65 years Study withdrawals: 0 *n* = 7 TZC + conventional treatment *n* = 6 conventional treatment	8 mg/kg/month TCZ 6 months	DLCO % mRSS	GI vs. GC: ↑DLCO % ↓ mRSS Changes from baseline: ↓ DLCO % ↓ mRSS

Abbreviations: ↑ = no significant increase; ↓ = no significant decrease; ↔ = no significant change. ↑* = significant increase; ↓* = significant decrease; *: indicates significant values (*p* < 0.05); PBO: placebo; TCZ: tocilizumab; SC: subcutaneous; mRSS: modified Rodnan scale; HAQ-DI Score: Health Assessment Questionnaire; FVC: forced vital capacity; DLCO: diffusion test for carbon monoxide; MMF: micofenolato mofetil.

**Table 6:** Number of adverse effects of biological drugs in the intervention group.

Adverse Effects	Biological Drugs				
	Tocilizumab	Belimumab	Riociguat	Abatacept	Romilkimab
Infections		8	1		
Cardiac	1				1
Gastrointestinal	1				
Respiratory				1	
Renal					
Vascular	1				
Neoplasms				1	
Musculoskeletal					
Nervous					
Endocrinologists					
Psychiatric	1				
Reproductive					
Blood and Lymphatics	1				
Dermatological					
Deaths					1

## Appendix A. Search Sequence Followed for Selection of Articles

#1 “Scleroderma, systemic sclerosis” [Mesh]

#2 “Scleroderma diffuse, diffuse cutaneous systemic sclerosis” [Mesh] #3 #1 OR #2

#4 “Therapeutics” [Mesh] OR “Tocilizumab” [Mesh] OR “Biological Therapy” [Mesh] OR “Antibodies, monoclonal” [Mesh] OR “Antibodies, monoclonal” [Mesh].

#5 #3 AND #4

The search was conducted in the following electronic databases: Medline (PubMed), Dialnet, and Cochrane Library Plus between September 2022 and December 2022. Languages were restricted to English and Spanish.

**Table A1:** Results of the methodological quality assessment of included studies—McMaster Critical Review Form for Quantitative Studies [28].

Study	Item																Total	%	Quality Score
	1	2	3	4	5	6	7	8	9	10	11	12	13	14	15	16			
Allanore et al. (2020) [30]	1	1	1	1	1	1	1	1	1	1	1	1	1	1	1	1	16	100	E
Gordon et al. (2018) [31]	1	1	1	1	1	1	1	1	0	0	0	0	1	0	1	1	11	68.7	G
Khanna Dinesh et al. (2018) [32]	1	1	1	1	1	1	1	1	0	0	1	1	1	1	1	1	14	87.5	VG
Khanna et al. (2020) [33]	1	1	1	1	1	1	1	1	1	1	0	1	1	1	1	1	15	93.7	E
Khanna et al. (2020) [34]	1	1	1	1	1	1	1	1	1	1	0	1	1	1	1	1	15	93.7	E
Shima et al. (2019) [35]	1	1	1	1	1	1	1	1	1	1	1	1	1	1	0	1	15	93.7	E

Abbreviations: 0 = not fulfilled criterion; 1 = fulfilled criterion; E = excellent; VG = very good; G = good. Item 1: study purpose; item 2: literature review; item 3: study design; item 4: blinding; item 5: sample description; item 6: sample size; item 7: ethics and consent; item 8: validity of outcomes; item 9: reliability of outcomes; item 10: intervention description; item 11: statistical significance; item 12: statistical analysis; item 13: clinical importance; item 14: conclusions; item 15: clinical implications; and item 16: study limitations.

**Table A2:** Results of the methodological quality assessment of included studies—PEDro scale [29].

Study	Item											Total	%	Quality Score
	1	2	3	4	5	6	7	8	9	10	11			
Allanore et al. (2020) [30]												11	100	E
Gordon et al. (2018) [31]												11	100	E
Khanna Dinesh et al. (2018) [32]												10	90.9	VG
Khanna et al. (2020) [33]												10	90.9	VG
Khanna et al. (2020) [34]												11	100	E
Shima et al. (2019) [35]												11	100	E

Abbreviations: Red = not fulfilled criterion; Green = fulfilled criterion; E = excellent; VG = very good; G = good; F = fair.
